# Inflammatory potential of the diet and self-rated quality of life in Italian adults

**DOI:** 10.3389/fnut.2026.1744444

**Published:** 2026-02-04

**Authors:** Francesca Giampieri, Justyna Godos, Giuseppe Caruso, Marco Antonio Olvera-Moreira, Fabrizio Furnari, Andrea Di Mauro, Irma Domínguez Azpíroz, Raynier Zambrano-Villacres, Evelyn Frias-Toral, Fabio Galvano, Giuseppe Grosso

**Affiliations:** 1 Department of Clinical Sciences, Università Politecnica Delle Marche, Ancona, Italy; 2 Research Group on Food, Nutritional Biochemistry and Health, Universidad Europea del Atlántico, Santander, Spain; 3 Joint Laboratory on Food Science, Nutrition, and Intelligent Processing of Foods, Polytechnic University of Marche, Italy, Universidad Europea del Atlántico Spain and Jiangsu University, China at Polytechnic University of Marche, Ancona, Italy; 4 Department of Biomedical and Biotechnological Sciences, University of Catania, Catania, Italy; 5 Departmental Faculty of Medicine, UniCamillus-Saint Camillus International University of Health Sciences, Rome, Italy; 6 IRCCS San Camillo Hospital, Venice, Italy; 7 Carrera de Medicina, Universidad Católica de Santiago de Guayaquil, Guayaquil, Ecuador; 8 Research Group on Food, Nutritional Biochemistry and Health, Universidade Internacional do Cuanza, Cuito, Bié, Angola; 9 Research Group on Food, Nutritional Biochemistry and Health, Universidad de La Romana, La Romana, Dominican Republic; 10 Dirección de Investigación, Universidad ECOTEC, Samborondón, Ecuador; 11 Escuela de Medicina, Universidad Espíritu Santo, Samborondón, Ecuador; 12 Division of Research, Texas State University, San Marcos, TX, United States

**Keywords:** diet quality, inflammation, mental health, physical health, quality of life

## Abstract

**Background:**

Dietary quality is widely acknowledged as a key factor in maintaining good health. Recommendations that promote plant-based eating patterns are largely grounded in evidence showing that dietary choices can modulate the immune function. In line with such a hypothesis, diet may be considered as a potential driver of persistent low-grade inflammation. Quality of life (QoL), on the other hand, serves as a broad indicator that encompasses both physical and psychological wellbeing.

**Aim:**

The purpose of this cross-sectional study was to examine the relationship between the inflammatory potential of the diet and QoL in a population sample of Italian adults.

**Design:**

A total of 1,936 participants completed a 110-item food frequency questionnaire to assess eating habits. The inflammatory potential of their diet was calculated using the dietary inflammatory score (DIS). Quality of life was measured with the Manchester Short Appraisal (MANSA).

**Results:**

Higher DIS values, reflecting a more pro-inflammatory diet, were linked to reduced likelihood of reporting high QoL (OR = 0.56; 95% CI: 0.40–0.78). Several specific domains of QoL, including general life satisfaction, social relationships, personal safety, satisfaction with cohabitation, physical health, and mental health, also showed significant associations with DIS.

**Conclusion:**

The findings suggest an association between the inflammatory potential of the diet and QoL.

## 1 Introduction

Diet quality represents one of the fundamental determinants of human health and significantly influences long-term health (1). A balanced diet may play a role in preventing the development of chronic diseases, such as type-2 diabetes, cancer, and cardiovascular disorders, supposedly by regulating the immune system and inflammatory processes (2, 3). In fact, diet is not only a source of energy and essential nutrients required for the sustenance and proper functioning of the body, but it also plays a crucial role as a direct modulator of systemic inflammatory processes (4). An activation of the immune responses as a chronic, subclinical phenomenon might lead to detrimental effects on overall health (5). This inflammatory process is characterized by consistently elevated levels of pro-inflammatory mediators and cytokines, which contribute over time to the progressive deterioration of biological systems (6). Chronic inflammation is implicated in several major health challenges including cardiovascular diseases, which represent the leading cause of mortality worldwide, metabolic disorders such as metabolic syndrome and obesity, and impairments in psychological and cognitive wellbeing, often linked to oxidative stress and neuroinflammation (7).

The qualitative and quantitative composition of meals may significantly influence the balance between pro- and anti-inflammatory mediators, with important long-term health consequences (8). Diets characterized by high consumption of simple sugars, refined carbohydrates and saturated fats may lead to increased production of pro-inflammatory mediators potentially contributing to the long-term deterioration of overall wellbeing (2). When sustained over time, this heightened inflammatory state promotes the development and progression of worsening health conditions (9). Furthermore, prolonged exposure to certain nutrients (i.e., trans fatty acids and simple sugars, among others) appears to negatively affect fundamental cellular and molecular processes, such as oxidative stress and the regulation of immune pathways, thereby amplifying the risk of developing complex pathological conditions and reducing the body’s overall resilience to physiological and environmental stressors (10). In contrast, dietary patterns characterized by a high intake of anti-inflammatory and antioxidant-rich foods, such as fruits, vegetables, legumes, whole grains and nuts, have been consistently associated with reduced levels of key inflammatory biomarkers, including C-reactive protein (CRP), various pro-inflammatory interleukins, and tumor necrosis factor-*α* (TNF-α) (11). These foods, being rich in plant-derived bioactive compounds, such as polyphenols, modulate inflammatory pathways and gut microbiota (12). Among the most extensively studied and globally recognized healthy dietary patterns is the Mediterranean diet (13), to which higher adherence not only ascertain better nutritional adequacy of the diet (14), but also represents a paradigmatic anti-inflammatory dietary model (15). Its primary components, including extra virgin olive oil, fruit and vegetables, legumes, nuts, and fatty fish, have been widely documented for their ability to reduce systemic inflammation and improve various markers of metabolic and cardiovascular health (16). A substantial body of scientific evidence has shown that high adherence to this dietary pattern is associated with a lower incidence of cardiovascular diseases (17), neurodegenerative disorders (18), and certain types of cancer (19), underlined by a low-grade subclinical chronic inflammation (20), partially mediated by gut microbiome dysbiosis (21).

Beyond their role in disease prevention, anti-inflammatory dietary patterns have been shown to positively affect not only physical health but also perceived wellbeing and overall QoL (22). Regular consumption of nutrient-dense foods contributes to improved physical and mental wellbeing, enhancing daily energy, cognitive function, and overall mood (23). These effects suggest that diet can act not only as a preventive factor against disease but also as a true modulator of perceived health, integrating clinical outcomes with psychological and psychosocial dimensions (24). Such benefits are particularly evident in older adults, for whom a balanced diet can help maintain autonomy, cognitive function, and overall QoL (25). Several indices have been developed to estimate the inflammatory potential of the diet based on the composition of pro- and anti-inflammatory elements (26–28). Among them, the Dietary Inflammatory Score (DIS) is an easy tool that could be considered both in a clinical setting or at population level to assess the inflammatory potential of the diet, but it has never been used to assess its association with QoL. Its easy calculation and the lack of previous studies on the topic provide the rationale for the choice of the DIS as a candidate tool to be applied at a broader level. The aim of this study was to investigate the relationship between the DIS and QoL in a representative sample of Italian adults in order to assess how adherence to pro- or anti-inflammatory diets may influence not only clinical and biological health parameters but also perceived wellbeing and individuals’ subjective perception of their own QoL.

## 2 Methods

### 2.1 Study design and population

The analyses for this cross-sectional study were carried out on a representative sample of 1936 men and women aged 18 years or older (29). Initially, 2,405 individuals were randomly selected and invited to participate in the investigation during the years 2014–2015 from the main districts of Catania, a metropolitan area located in southern Italy. The recruitment process achieved an 85% response rate, yielding a final sample of 2044 respondents. After excluding unreliable dietary intakes (i.e., <1,000 or >6,000 kcal/day), identified through case-by-case verification, and missing data, a final sample of 1,936 was considered as the current analytical sample. All study procedures were conducted in accordance with the ethical principles outlined in the World Medical Association’s Declaration of Helsinki (1989).

### 2.2 Background data

The background information collected for each participant included sex, age at the time of recruitment, highest level of education attained, current occupational status (or, in the case of retired individuals, the main occupation held during their working life), level of physical activity, and smoking habits. Educational attainment was classified into three categories: (i) low (primary or secondary school), (ii) medium (high school), and (iii) high (university degree). Occupational status was categorized as (i) unemployed, (ii) low, (iii) medium, and (iv) high. The level of physical activity was assessed through the International Physical Activity Questionnaire (IPAQ) and subsequently grouped into three categories: (i) low, (ii) moderate, and (iii) high (30). Smoking behavior was classified as (i) never smoker, (ii) former smoker, or (iii) current smoker.

### 2.3 Health outcome data

Systematic information on participants’ health status was collected, with particular focus on cardiometabolic conditions, including obesity, hypertension, type 2 diabetes, and dyslipidemia. Specifically, data on obesity, hypertension, type-2 diabetes, and dyslipidemia were recorded and categorized in a binary format (yes/no). This approach allowed a clear assessment of the prevalence of these conditions within the study sample. Obesity was defined based on body mass index (BMI) ≥ 30 kg/m^2^ according to international guidelines, while hypertension, diabetes, and dyslipidemia were identified through self-reported medical diagnoses or current use of specific medications for these conditions. This classification enabled the evaluation of associations between the dietary inflammatory potential, measured via DIS, and the presence of major chronic health conditions.

### 2.4 Dietary assessment

Dietary habits were assessed through the administration of a 110-item food frequency questionnaire referring to the dietary intakes of the previous 6 months, previously validated in the Sicilian population (31, 32). The consumption of seasonal foods was recorded for the period in which they were available and subsequently adjusted to reflect their proportional annual intake. Based on the reported food consumption, the energy content as well as macro- and micronutrient intake were calculated using the food composition tables provided by the Council for Research in Agriculture and Analysis of Agricultural Economy (CREA) (33). For each participant, individual food consumption (expressed in milliliters or grams) was estimated according to standard portion sizes and converted to daily intake. These values were then matched with the databases to obtain average energy content and nutrient composition per 100 mL or grams of each food item.

### 2.5 Dietary inflammatory score

The inflammatory potential of the diet was estimated by applying a validated score including 18 components, including leafy greens and cruciferous vegetables, legumes, refined grains, and starchy vegetables, apples and berries, deep yellow or orange vegetables and fruit, tomatoes, other fruits, and real fruit juices, other vegetables, added sugars, red and organ meats, processed meats, fish, poultry, high-fat dairy, low-fat dairy and tea, nuts, and other fats (34). For score calculation, intake of each food group was standardized to the study population distribution by converting values to z-scores. These standardized intakes were multiplied by predefined inflammatory weights derived from regression coefficients relating each food group to the composite inflammation biomarker score in the DIS development cohort. Weighted food-group values were then summed to generate an overall DIS value for each participant. The DIS is a continuous score, with higher values indicating a more pro-inflammatory diet and lower (more negative) values indicating a more anti-inflammatory dietary pattern. Because the DIS is population-standardized, the absolute score range is sample-dependent; however, scores are typically centered around zero, with positive and negative values reflecting relative dietary inflammatory potential within the study population.

### 2.6 Self-rated QoL

The Manchester Short Appraisal (MANSA) was used to assess self-rated QoL (35). In particular, the scale comprises 12 subjective items with a seven-point Likert scale (from “could not be worse” to “could not be better”) and four yes/no questions related to objective aspects of social life. The scale assesses satisfaction with life as a whole and across several specific domains (including employment, financial situation, friendships, leisure activities, accommodation, personal safety, people living in household/living alone, sex life, relationship with family, and physical and mental health). The maximum points achievable is 84, with higher points reflecting higher QoL. For the purposes of this study, satisfaction with overall QoL was arbitrarily defined as being in the highest quartile of the MANSA score (>70 points).

### 2.7 Statistical analysis

Overall and individual domains of QoL represented the main outcomes of interest for this study. Categorical variables are presented as absolute numbers and percentages, while continuous variables are reported as means with standard deviations. Group differences for categorical and continuous variables were assessed using the Chi-square test and ANOVA, respectively, or the Kruskal–Wallis test for non-normally distributed variables. Logistic regression analyses were conducted to estimate odds ratios (ORs) and 95% confidence intervals (CIs) for the association between tertiles of DIS score and QoL. Associations were examined using multivariable logistic regression models adjusted for sex, age, educational level, occupational level, smoking status, physical activity, and health status (including obesity, hypertension, type-2 diabetes, and dyslipidemia). A significance level of 0.05 was applied, and all *p*-values reported are two-sided. Statistical analyses were performed using SPSS version 27 (SPSS Inc., Chicago, IL, United States).

## 3 Results

The mean DIS score of the study sample was −0.058 (range −16.487 to 9.400), with mean scores in the tertiles of −2.151 ± 1.828, 0.1667 ± 0.404, and 1.931 ± 1.118. Participants were equally distributed across age groups, with 58.5% of participants being female and 61.7% non-smokers (Table 1). All participants were also equally distributed across categories of education and occupational level, with the majority reporting medium physical activity levels (Table 1). Obesity accounted for 17.6% of individuals, while hypertension resulted in much higher prevalence (Table 1). Regarding the background characteristics of the study sample across DIS tertiles, the most notable differences were observed for age groups, smoking and physical activity, with a higher proportion of younger, smoker, and medium physically active participants belonging to the group with higher DIS scores, reflecting a more pro-inflammatory diet (Table 1).

**Table 1:** Background characteristics of the study sample by tertiles of DIS score.

	DIS tertiles				*P*-value
	Total (*n* = 1936)	T1 (*n* = 664)	T2 (*n* = 647)	T3 (*n* = 625)	
Age groups, n (%)					0.031
<39	683 (35.3)	226 (34.0)	206 (31.8)	251 (40.2)	
40–59	681 (35.2)	234 (35.2)	240 (37.1)	207 (33.1)	
>59	572 (29.5)	204 (30.7)	201 (31.1)	167 (26.7)	
Sex, n (%)					0.759
Male	804 (41.5)	273 (41.1)	264 (40.8)	267 (42.7)	
Female	1,132 (58.5)	391 (58.9)	383 (59.2)	358 (57.3)	
Smoking status, n (%)					0.018
Non-smoker	1,195 (61.7)	428 (64.5)	400 (61.8)	367 (58.7)	
Current smoker	465 (24.0)	149 (22.4)	140 (21.6)	176 (28.2)	
Former smoker	276 (14.3)	87 (13.1)	107 (16.5)	82 (13.1)	
Educational level, n (%)					0.713
Low	697 (36.0)	243 (36.6)	237 (36.6)	217 (34.7)	
Medium	720 (37.2)	235 (35.4)	241 (37.2)	244 (39.0)	
High	519 (26.8)	186 (28.0)	169 (26.1)	164 (26.2)	
Occupational level, n (%)					0.119
Unemployed	461 (27.8)	243 (36.6)	237 (36.6)	217 (34.7)	
Low	266 (16.0)	235 (35.4)	241 (37.2)	244 (39.0)	
Medium	440 (26.5)	186 (28.0)	169 (26.1)	164 (26.2)	
High	491 (29.6)	180 (29.7)	140 (24.4)	141 (29.5)	
Physical activity level, n (%)					0.047
Low	329 (19.0)	117 (20.1)	105 (18.5)	107 (18.5)	
Medium	856 (49.5)	259 (44.5)	292 (51.5)	305 (52.7)	
High	543 (31.4)	206 (35.4)	170 (30.0)	167 (28.8)	
Health status, n (%)					
Obesity	317 (17.6)	96 (15.4)	107 (17.7)	114 (20.0)	0.112
Hypertension	976 (50.4)	326 (49.1)	347 (53.6)	303 (48.5)	0.130
Diabetes	146 (7.5)	53 (8.0)	51 (7.9)	42 (6.7)	0.638
Dislipidemia	356 (18.4)	135 (20.3)	109 (16.8)	112 (17.9)	0.248

DIS, dietary inflammatory score; T, tertile; SD, standard deviation.

The general dietary intakes by tertiles of DIS is presented in Table 2. The sample consumed a fairly adequate amount of daily fruit and vegetables, high intake of cereals (although only a small proportion whole grains), high intake of nuts, and relatively low alcohol (Table 2). Red meat is also consumed within recommended ranges while processed meat intake was relatively high (Table 2). Total food consumption and nutrient intake reflected the rationale behind the calculation of the DIS. Participants with lower DIS scores tended to consume higher amounts of fruits (258.7 ± 208.0 g/d vs. 645.8 ± 446.9 g/d), vegetables (196.3 ± 115.8 g/d vs. 378.1 ± 244.1 g/d), whole grains (30.8 ± 58.6 g/d vs. 53.3 ± 65.5 g/d), legumes (34.3 ± 43.0 g/d vs. 48.9 ± 70.0 g/d), dairy products (179.2 ± 162.0 g/d vs. 227.0 ± 210.4 g/d), fish (61.7 ± 73.0 g/d vs. 87.7 ± 111.6 g/d), nuts (15.8 ± 20.5 g/d vs. 29.9 ± 68.2 g/d), and alcohol (6.2 ± 11.1 mL/d vs. 9.4 ± 12.8 mL/d) (Table 2). Conversely, participants with higher DIS scores showed a more frequent consumption of refined grains (241.4 ± 141.9 g/d vs. 144.1 ± 105.2 g/d), red meat (40.8 ± 28.3 g/d vs. 30.1 ± 25.7 g/d), processed meat (23.9 ± 27.8 g/d vs. 12.9 ± 17.9 g/d), sweets and desserts (70.1 ± 75.5 g/d vs. 55.7 ± 61.7 g/d), and sugar-sweetened beverages (80.8 ± 164.4 mL/d vs. 20.8 ± 51.6 mL/d) (Table 2).

**Table 2:** Mean consumption (and standard deviation) or major food groups by tertiles of DIS score.

	DIS tertiles				*P*-value
	Total (*n* = 1936)	T1 (*n* = 664)	T2 (*n* = 647)	T3 (*n* = 625)	
		Mean (SD)			
Fruit (g/d)	414.9 (354.0)	645.8 (446.9)	328.8 (207.1)	258.7 (208.0)	<0.001
Vegetables (g/d)	273.5 (187.4)	378.1 (244.1)	240.7 (114.2)	196.3 (115.8)	<0.001
Potatoes (g/d)	26.5 (30.9)	25.7 (27.5)	23.8 (32.2)	30.2 (32.6)	0.001
Cereals (g/d)	221.1 (137.6)	197.4 (118.5)	196 (125.1)	272.3 (154)	<0.001
Refined grains (g/d)	183.5 (128.7)	144.1 (105.2)	167.9 (117.3)	241.4 (141.9)	<0.001
Whole grains (g/d)	37.6 (57.5)	53.3 (65.5)	28.2 (42.6)	30.8 (58.6)	<0.001
Legumes (g/d)	39.0 (51.5)	48.9 (70.0)	33.5 (31.2)	34.3 (43.0)	<0.001
Total meat (g/d)	71.3 (40.0)	70.1 (43.2)	69.4 (36.5)	74.8 (39.9)	0.031
Red meat (g/d)	34.5 (26.5)	30.1 (25.7)	32.9 (24.2)	40.8 (28.3)	<0.001
Processed total meat (g/d)	17.2 (21.5)	12.9 (17.9)	15.2 (15.6)	23.9 (27.8)	<0.001
Dairy (g/d)	199.6 (181.2)	227 (210.4)	191.2 (162.3)	179.2 (162)	<0.001
Fish (g/d)	69.6 (84.1)	87.7 (111.6)	58.6 (52.5)	61.7 (73)	<0.001
Eggs (n eggs)	2.4 (4.7)	2.5 (4.7)	2.4 (4.8)	2.5 (4.9)	0.984
Sweets and dessert (g/d)	62.2 (69.6)	55.7 (61.7)	61.4 (70.7)	70.1 (75.5)	0.001
Sweet beverages (ml/d)	42.4 (110.3)	20.8 (51.6)	27.5 (74.2)	80.8 (164.4)	<0.001
Olive oil (n spoons)	7.08 (3.1)	7.5 (3.1)	7.1 (3)	6.6 (3.2)	<0.001
Nuts (g/d)	22.6 (45.0)	29.9 (68.2)	21.7 (28.1)	15.8 (20.5)	<0.001
Alcohol (ml/d)	7.6 (11.9)	9.4 (12.8)	7 (11.6)	6.2 (11.1)	<0.001

DIS, dietary inflammatory score; T, tertile; SD, standard deviation.

Findings consistent with food group consumption were also found concerning the distribution of selected macro- and micronutrients across DIS tertiles (Table 3). Participants with higher DIS scores had a greater total energy intake (2,225 ± 905.1 kcal/d vs. 2226.5 ± 868.3 kcal/d), a higher consumption of total lipids (65.0 ± 33.3 g/d vs. 65.3 ± 29.1 g/d), particularly saturated fats (26.3 ± 13.2% vs. 24.7 ± 12.0%), and a lower intake of omega-3 fatty acids (total omega-3: 1.6 ± 0.8% vs. 2.0 ± 1.1%; seafood omega-3: 0.5 ± 0.5% vs. 0.7 ± 0.8%), fiber (29.1 ± 15.4 g/d vs. 42.3 ± 22.5 g/d), vitamins (vitamin A: 689.3 ± 325.9 μg/d vs. 1199.8 ± 566.2 μg/d; vitamin C: 115.4 ± 73.1 mg/d vs. 241.3 ± 148.6 mg/d; vitamin E: 8.4 ± 4.3 mg/d vs. 10.6 ± 4.6 mg/d), and minerals (calcium: 711.7 ± 282.3 mg/d vs. 930.3 ± 380.5 mg/d; potassium: 3317.0 ± 1657.9 mg/d vs. 4849.3 ± 2239.5 mg/d; magnesium: 363.9 ± 124.5 mg/d vs. 455.5 ± 154.3 mg/d) (Table 3).

**Table 3:** Distribution of selected macro- and micronutrients by tertiles of DIS score.

	DIS tertiles				*P*-value
	Total (*n* = 1936)	T1 (*n* = 664)	T2 (*n* = 647)	T3 (*n* = 625)	
		Mean (SD)			
Energy (kcal/d)	2,113.9 (818.1)	2,226.5 (868.3)	1,891.1 (604.1)	2,225 (905.1)	<0.001
Energy (KJ/d)	8,572. 7 (3389.6)	8990.4 (3636.5)	7647.3 (2478.8)	9087.1 (3725.4)	<0.001
Proteins (g/d)	88.5 (38.5)	95.8 (45.2)	79.2 (24.9)	90.3 (40.4)	<0.001
Fats (g/d)	62.5 (27.9)	65.3 (29.1)	57.3 (19)	65 (33.3)	<0.001
Cholesterol (mg/d)	199.8 (111.6)	203.6 (131.1)	182.6 (74.1)	213.9 (119)	<0.001
Saturated fats (%)	24.5 (11.4)	24.7 (12)	22.7 (8.4)	26.3 (13.2)	<0.001
Monounsaturated fats (%)	26.3 (11.0)	27.2 (10.8)	24.4 (8.0)	27.5 (13.4)	<0.001
Polyunsaturated fats (%)	11.5 (5.8)	12.1 (6.0)	10.3 (4.3)	12.1 (6.9)	<0.001
Total omega-3 (%)	1.7 (0.8)	2 (1.1)	1.6 (0.6)	1.6 (0.8)	<0.001
Seafood omega-3 (%)	0.5 (0.6)	0.7 (0.8)	0.5 (0.4)	0.5 (0.5)	<0.001
Plant omega-3 (%)	1.7 (0.5)	1.3 (0.7)	1.1 (0.4)	1.1 (0.4)	<0.001
Carbohydrates (g/d)	308.17 (127.8)	321.1 (131.6)	272.1 (104.4)	331.8 (137.5)	<0.001
Total fiber (g/d)	33.5 (18.3)	42.3 (22.5)	28.6 (11.7)	29.1 (15.4)	<0.001
Vitamin A retinol eq. (μg/d)	898.4 (473.7)	1,199.8 (566.2)	790.9 (305.6)	689.3 (325.9)	<0.001
Vitamin C (mg/d)	166.7 (118.1)	241.3 (148.6)	139.6 (70.4)	115.4 (73.1)	<0.001
Vitamin E (mg/d)	9.04 (4.01)	10.6 (4.6)	8 (2.6)	8.4 (4.3)	<0.001
Vitamin D (μg/d)	5.8 (6.6)	7 (8.4)	4.9 (4.1)	5.6 (6.4)	<0.001
Vitamin B1 (mg/d)	1.7 (0.8)	1.9 (0.9)	1.7 (0.8)	1.7 (0.8)	<0.001
Vitamin B2 (mg/d)	2.2 (0.9)	2.5 (1)	2.1 (0.9)	2.1 (0.9)	<0.001
Vitamin B3 (mg/d)	22.1 (8.3)	24.4 (9.6)	20.2 (6.1)	21.8 (8.2)	<0.001
Vitamin B6 (mg/d)	2.5 (0.8)	2.9 (1.0)	2.3 (0.7)	2.4 (0.8)	<0.001
Vitamin B9 (μg/d)	392. 3 (164.8)	469.6 (191.9)	359.3 (130.8)	346.1 (135)	<0.001
Vitamin B12 (μg/d)	6.9 (7.3)	8 (10.9)	5.8 (3.1)	6.9 (5.3)	<0.001
Sodium (mg/d)	2,930. 7 (1210.2)	2,819.5 (1157.8)	2,706.4 (923.5)	3,281.1 (1430.5)	<0.001
Potassium (mg/d)	3,857.8 (1874.3)	4,849.3 (2239.5)	3,362.7 (1087)	3,317 (1657.9)	<0.001
Zinc (mg/d)	12.2 (4.5)	13.7 (5.4)	11.2 (3.6)	11.7 (4.1)	<0.001
Calcium (mg/d)	799.8 (331.1)	930.3 (380.5)	753.5 (277.5)	711.7 (282.3)	<0.001
Iron (mg/d)	15.3 (5.8)	17.5 (6.8)	14 (4.7)	14.5 (5.2)	<0.001
Magnesium (mg/d)	394.3 (139.3)	455.5 (154.3)	362.2 (114.7)	363.9 (124.5)	<0.001
Selenium (μg/d)	103.9 (46.8)	104.5 (48.5)	94.6 (40.5)	113.6 (49.2)	<0.001

DIS, dietary inflammatory score; T, tertile; SD, standard deviation.

The overall mean MANSA score was 62.6 ± 9.5 (range 31 to 84). The association between DIS and QoL and its individual components are shown in Table 4. Higher DIS values, reflecting a more pro-inflammatory diet, were associated with a higher likelihood of reporting low overall QoL (adjusted OR = 1.77; 95% CI: 1.27–2.46). In addition, several specific domains of QoL showed significant associations with DIS, including overall life satisfaction (adjusted OR = 1.36; 95% CI: 1.02–1.80), social relationships as reflected by the amount and quality of friendships (adjusted OR = 1.48; 95% CI: 1.13–1.95), personal safety (adjusted OR = 1.34; 95% CI: 1.01–1.76), satisfaction with cohabitation (adjusted OR = 1.45; 95% CI: 1.09–1.94), physical health (adjusted OR = 1.65; 95% CI: 1.25–2.18), and mental health (adjusted OR = 1.68; 95% CI: 1.25–2.24).

**Table 4:** Association between overall QoL and individual domain satisfaction of the study participants and tertiles of DIS score.

	DIS tertiles, OR (95% CI)*		
	T1	T2	T3
Overall QoL			
Unadjusted	1	1.36 (1.05, 1.76)	1.54 (1.18, 2.00)
Adjusted	1	1.42 (1.05, 1.91)	1.77 (1.27, 2.46)
Overall life satisfaction			
Unadjusted	1	1.14 (0.91, 1.24)	1.85 (0.95, 1.48)
Adjusted	1	1.18 (0.90, 1.54)	1.36 (1.02, 1.80)
Job (when having one)			
Unadjusted	1	1.16 (0.91, 1.46)	1.21 (0.95, 1.54)
Adjusted	1	1.34 (1.01, 1.77)	1.43 (1.06, 1.92)
Financial situation			
Unadjusted	1	1.20 (0.92, 1.56)	1.23 (0.94, 1.61)
Adjusted	1	1.55 (0.85, 1.56)	1.44 (1.03, 2.01)
Amount and quality of friends			
Unadjusted	1	1.11 (0.90, 1.38)	1.43 (1.15, 1.80)
Adjusted	1	1.11 (0.86, 1.44)	1.48 (1.13, 1.95)
Leisure activities			
Unadjusted	1	1.03 (0.81, 1.31)	1.56 (0.90, 1.47)
Adjusted	1	1.04 (0.78, 1.38)	1.38 (1.02, 1.88)
Housing			
Unadjusted	1	1.00 (0.81, 1.25)	1.18 (0.95, 1.47)
Adjusted	1	0.95 (0.73, 1.22)	1.23 (0.94, 1.61)
Accused of a crime			
Unadjusted	1	1.31 (0.29, 5.83)	1.88 (0.35, 10.34)
Adjusted	1	1.15 (0.18, 7.34)	2.53 (0.020, 31.37)
Personal safety			
Unadjusted	1	1.15 (0.93, 1.44)	1.28 (1.03, 1.60)
Adjusted	1	1.17 (0.91, 1.53)	1.34 (1.01, 1.76)
People with whom individual lives			
Unadjusted	1	1.15 (0.91, 1.45)	1.29 (1.03, 1.63)
Adjusted	1	1.13 (0.86, 1.50)	1.45 (1.09, 1.94)
Sex life			
Unadjusted	1	1.15 (0.92, 1.43)	1.38 (0.91, 1.42)
Adjusted	1	1.25 (0.96, 1.62)	1.36 (1.03, 1.79)
Relationship with family			
Unadjusted	1	1.15 (0.88, 1.40)	1.18 (0.93, 1.49)
Adjusted	1	1.06 (0.80, 1.41)	1.19 (0.89, 1.59)
Physical health			
Unadjusted	1	1.30 (1.04, 1.62)	1.35 (1.08, 1.68)
Adjusted	1	1.31 (1.01, 1.70)	1.65 (1.25, 2.18)
Mental health			
Unadjusted	1	1.37 (1.08, 1.73)	1.58 (1.25, 1,99)
Adjusted	1	1.38 (1.05, 1.83)	1.68 (1.25, 2.24)

*Analysis adjusted for sex, age, educational level, occupational level, smoking status, physical activity, and health status (including obesity, hypertension, type-2 diabetes, and dyslipidemia). DIS, dietary inflammatory score; T, tertile; SD, standard deviation; QoL, quality of life.

## 4 Discussion

The findings of this study confirm a significant association between higher dietary inflammatory potential, as quantified by the DIS score, and worse QoL in Italian adults. Specifically, the impact extends across several QoL domains, including overall life satisfaction, physical and mental health, social relationships, and perceived personal safety. Notably, the association between diet and QoL was not mediated by metabolic conditions (such as obesity, hypertension, type-2 diabetes, and dyslipidemia) since there were no significant differences in distribution of such variables between individuals with different DIS tertiles. These observations can be interpreted in light of the pathophysiological mechanisms linking low-grade chronic inflammation to adverse psycho-physical health outcomes (20). Previous studies have demonstrated that diets characterized by a high intake of pro-inflammatory foods promote the production of pro-inflammatory cytokines (IL-6, TNF-*α*, CRP), oxidative stress, and the activation of neuroendocrine pathways involved in stress response (36). On the contrary, preclinical evidences highlight many components of a healthy diet with both prebiotic and biotic properties, including (poly) phenols (37) fermentable dietary fibers and probiotics (38), like Lactobacillus species, which may reduce chronic inflammation via modulation of gut microbiota (39), and as more recently suggested via oral microbiota (40). These processes have been associated with depressive symptoms, fatigue, reduced vitality, and cognitive impairment, all of which contribute to a negative perception of subjective wellbeing (41). Our results are supported by previous literature documenting the association between systemic inflammatory markers and reduced mental health, psychological wellbeing, and physical performance (42). A systematic review and meta-analysis investigating inflammatory markers in individuals with clinically significant depression, anxiety, or post-traumatic stress disorder (PTSD) found that each of these conditions is associated with an inflammatory response, even in the absence of comorbid mental or physical health problems typically linked to inflammation (43). Also, a systematic review reported consistency in a significant inverse relationship between DII scores and overall QoL and/or its subscales across various populations, including patients with asthma, osteoarthritis, hemodialysis, multiple sclerosis, obese women, and healthy individuals (44). These studies strengthen the hypothesis that the inflammatory potential of the diet represents a modifiable determinant of QoL, suggesting that nutritional strategies aimed at lowering dietary inflammatory load, such as implementation of Mediterranean diet (45), may serve as both preventive and therapeutic approaches not only for the reduction of chronic disease risk but also for the promotion of overall wellbeing and QoL (46).

Analyzing the dietary composition, it emerged that participants with lower DIS scores tended to consume greater amounts of fruits, vegetables, whole grains, legumes, dairy products, fish, nuts, and alcohol. Conversely, individuals with higher DIS scores consumed refined grains, red and processed meat, sweets, and sugar-sweetened beverages more frequently. This differentiation suggests that a diet rich in plant-based foods rich in fiber and phytochemical, such as (poly) phenols, as well as rich in sources of unsaturated fats is associated with a more favorable inflammatory profile, whereas the intake of foods high in simple sugars and saturated fats promotes a pro-inflammatory state (47), consistent with previous evidence (48). In parallel, the analysis of macro- and micronutrients showed that higher DIS values were associated with greater total energy intake and a higher intake of lipids, particularly saturated fats, alongside a reduced intake of omega-3 fatty acids, fiber, vitamins, and minerals (49). These findings further support the link between diet quality, inflammation, and potential effects on QoL, suggesting that nutritional balance plays a key role in modulating the inflammatory state and, consequently, subjective wellbeing (50). Moreover, chronic low-grade inflammation may worsen overall physical health by contributing to chronic pain conditions, reduced motor functionality, and increased vulnerability to metabolic and cardiovascular diseases (51). These effects, in addition to directly lowering physical QoL, may indirectly impair social and relational dimensions, limiting active participation in community life and negatively affecting interpersonal satisfaction and perceived personal security (52). It is important to emphasize that these physiological impairments extend their impact beyond objective physical health, exerting profound effects on health-related QoL (53). Functional limitations arising from chronic inflammatory states can hinder the performance of daily activities, compromise independence, and increase reliance on social support networks, as well as negatively affect social and relational domains by limiting active participation in community and occupational activities, reducing opportunities for interpersonal interaction, and diminishing the perception of social connectedness (54). Consequently, chronic inflammation may generate a cycle in which reduced physical functioning leads to lower social participation, which in turn can exacerbate psychological stress and perceived vulnerability, highlighting the bidirectional interplay between physiological and psychosocial determinants of wellbeing (55).

Finally, when considering background characteristics, participants with higher DIS values were more likely to be smokers and to engage in lower levels of physical activity. This association suggests that the effect of diet on inflammatory status and, consequently, on QoL, cannot be considered in isolation but should be interpreted within a broader behavioral context (56). It is well established that both cigarette smoking and physical inactivity promote the activation of inflammatory pathways and oxidative stress, thereby contributing to increased systemic levels of pro-inflammatory cytokines (57). In particular, tobacco smoke is known to be a significant source of reactive oxygen and nitrogen species (ROS and RNS), which can damage cellular and sub-cellular components such as lipids, proteins, and nucleic acids (58). A substantial body of evidence has highlighted that smoking-induced ROS and the resulting oxidative stress play a central role in the activation of inflammatory processes and in the mechanisms of carcinogenesis (59).

The co-occurrence of these unhealthy behaviors with a pro-inflammatory dietary pattern may therefore exert a synergistic effect, amplifying the risk of impaired physical and mental health and worsening perceived QoL (60). These findings underscore the importance of preventive approaches including interventions aimed at smoking cessation and the promotion of physical activity (61). Despite these encouraging findings, important gaps remain in the scientific literature, particularly regarding studies specifically focused on the relationship between the inflammatory potential of the diet and self-perceived quality of life in the general population, and even more so within cultural and dietary contexts such as Italy (62). Given the central role of food in the Italian lifestyle and the growing interest in promoting health through integrated nutritional and psychosocial approaches, exploring this link may provide valuable insights for public health strategies.

The study has several strengths. Key strengths include the use of validated instruments to assess both dietary intake and quality of life, enhancing the reliability and comparability of the findings despite reliance on self-reported data. The application of an evidence-based dietary inflammatory score allows a biologically plausible evaluation of the relationship between diet and quality of life. Analyses accounted for a wide range of sociodemographic, lifestyle, and health-related confounders, reducing the potential for residual confounding. Additionally, the focus on an Italian adult population provides novel context-specific evidence in a Mediterranean setting, and the assessment of multiple QoL domains offers a comprehensive perspective on wellbeing.

The findings of this study should be interpreted in light of some limitations. First, the cross-sectional design raises the possibility of reverse causality. Second, the use of self-reported dietary intake and QoL measures may introduce recall bias. However, the instruments used were all validated and represent the gold standard for such types of studies. Third, although we adjusted for several factors that could reasonably affect the associations, residual confounding for unmeasured variables may still occur. Finally, because the study was conducted on a representative sample of the Southern Italian population, the generalizability of the results to other populations may be limited.

In conclusion, the inflammatory properties of an individual’s diet could represent a key underlying mechanism explaining the observed relationships between dietary patterns and overall health outcomes. Diets with higher pro-inflammatory potential may contribute to an increased risk of chronic conditions, while dietary patterns rich in anti-inflammatory components could support the maintenance of physiological balance, enhance wellbeing, and improve long-term health. These findings suggest that promoting dietary patterns with lower inflammatory potential may represent a feasible strategy to support overall wellbeing in the general population, alongside established lifestyle recommendations. From a clinical and public health perspective, consideration of the inflammatory properties of the diet could complement existing nutritional guidance aimed at improving patient-centered outcomes such as quality of life. Future research should prioritize longitudinal studies to clarify temporal and causal relationships and intervention trials to determine whether reducing dietary inflammatory potential leads to meaningful improvements in quality of life. Additionally, studies integrating objective inflammatory biomarkers and exploring potential effect modification by age, sex, or clinical conditions are warranted to better elucidate underlying mechanisms and identify populations most likely to benefit. Overall, understanding the inflammatory impact of diet provides valuable insights for developing nutritional strategies aimed at promoting optimal health and preventing disease across diverse populations.

## Data Availability

The original contributions presented in the study are included in the article/supplementary material, further inquiries can be directed to the corresponding author.
